# Optical Coherence Tomography Angiography in Detecting Subclinical Changes in Glaucoma Suspects: A Scoping Review

**DOI:** 10.7759/cureus.88000

**Published:** 2025-07-15

**Authors:** Roberto A Hidalgo Ramos, Sebastián Dufner Krieger, Marcelo Ortiz Meneses

**Affiliations:** 1 Medicine, Universidad de Costa Rica, San José, CRI

**Keywords:** early detection, glaucoma suspects, microvascular changes, oct angiography, optical coherence tomography (oct), vessel density

## Abstract

Optical coherence tomography angiography (OCTA) offers a promising approach for detecting subclinical microvascular changes in glaucoma suspects before structural or functional damage becomes apparent. This scoping review synthesizes evidence from 11 studies (2014-2025) to evaluate OCTA’s effectiveness in identifying early glaucomatous alterations. Findings consistently demonstrate reduced vessel density (VD) in glaucoma suspects compared to healthy controls, with whole-image VD averaging 51.3% versus 55.5% (P<0.001), respectively. OCTA-derived parameters, particularly peripapillary and macular VD, showed diagnostic accuracy (area under the receiver-operating characteristic curve (AUROC): 0.70-0.84) comparable to structural OCT metrics like retinal nerve fiber layer thickness. Notably, microvascular asymmetry and deep-layer capillary dropout emerged as early markers, often preceding visual field defects. Longitudinal data revealed faster VD decline in preperimetric glaucoma (−2.23%/year) than in healthy eyes (0.29%/year), highlighting OCTA's potential for monitoring progression. Despite methodological heterogeneity, OCTA complements structural assessments, providing actionable insights for early glaucoma detection. Standardization of protocols and longitudinal validation are needed to optimize clinical utility. These findings support OCTA's role in enhancing risk stratification for glaucoma suspects.

## Introduction and background

Introduction

Glaucoma is a subacute optic neuropathy that is considered one of the major causes of irreversible blindness across the globe. It is defined by the impairment of the optic nerve, including the loss of the retinal ganglion cells and their axons [1-4]. Although the primary risk factor in the development and prognosis of high intraocular pressure (IOP) is elevated, vascular factors are more important in the development and progression of the disease [1-7]. The most important aspect is found in early detection, in that visual field (VF) loss is one of the standard diagnostic criteria that frequently becomes apparent when significant loss of retinal ganglion cells (RGCs) has already occurred [1, 2, 4, 5]. Glaucoma suspects are usually individuals who have been identified as having risk factors but do not yet exhibit optic nerve damage or classical VF defects [1-4, 6, 8]. Preperimetric glaucoma (PPG), in turn, is used to characterize patients with early optic nerve damage without detectable VF loss [1, 4]. Optical coherence tomography (OCT) enables structural assessment through objective measurements of retinal nerve fiber layer (RNFL) and ganglion cell complex (GCC) thickness, which are useful in glaucoma monitoring but do not reflect early vascular involvement [3, 4]. In contrast, optical coherence tomography angiography (OCTA) offers a non-invasive, dye-free imaging technique that uses motion contrast to generate three-dimensional, depth-resolved images of the retinal and optic nerve head microvasculature [1-4, 6, 7]. This technology enables quantitative measurement of vessel density (VD) in the optic nerve head, peripapillary region, and macular area. Early identification of subclinical microvascular alterations, particularly in PPG, is clinically important as it enhances understanding of glaucoma pathophysiology and facilitates earlier diagnosis and intervention. This review primarily aims to assess the effectiveness of OCTA in detecting subclinical microvascular changes in glaucoma suspects.

Objectives

Primary Objective

To assess the effectiveness of OCTA in detecting subclinical microvascular changes in glaucoma suspects.

Secondary Objectives

To summarize VD, perfusion parameters, and capillary dropout findings across studies.

## Review

Methodology

Research Question in Patient/Problem, Intervention, Comparison, and Outcome (PICO) Framework

The research question was developed using the PICO (Patient/Problem, Intervention, Comparison, and Outcome) framework. The population comprised adults identified as glaucoma suspects, defined as individuals with suspicious optic nerve head appearance or elevated IOP but without detectable VF defects. The intervention evaluated was the use of OCTA for imaging and assessing the microvasculature of the retina and optic nerve head. While not essential, some studies included comparators such as healthy individuals or alternative diagnostic methods, including standard structural OCT or VF testing. The outcomes of interest focused on subclinical microvascular changes, particularly reductions in VD, perfusion deficits, and evidence of capillary dropout. Based on this framework, the final research question guiding this scoping review was: In adults identified as glaucoma suspects or with PPG, how effective is OCTA in detecting subclinical microvascular changes compared to standard diagnostic approaches or healthy controls?

Eligibility Criteria

Studies were eligible for inclusion if they involved human adults (aged 18 or older) identified as glaucoma suspects or individuals diagnosed with PPG. Eligible studies utilized OCTA to assess retinal or optic nerve head microvasculature and reported subclinical findings such as VD, perfusion deficits, capillary dropout, or changes in the foveal avascular zone (FAZ). Observational designs - specifically cross-sectional, cohort, or case-control studies - were included, as well as comparative studies incorporating healthy control groups. Only peer-reviewed journal articles published in English between January 2014 and the present, and indexed in PubMed, the Cochrane Library, ScienceDirect, or Google Scholar, were considered.

Exclusion criteria encompassed animal studies, in vitro research, and studies that focused exclusively on patients with established glaucoma (e.g., primary open-angle glaucoma) without addressing suspects or preperimetric cases. Studies lacking OCTA imaging or those not reporting quantitative OCTA outcomes, such as VD or perfusion metrics, were excluded. Additionally, reviews, meta-analyses, editorials, conference abstracts, case reports, and letters were excluded. Non-English publications, duplicate records, or overlapping datasets were also removed, with only the most comprehensive or recent version retained for analysis.

Information Sources and Source Strategy

A systematic search was conducted across four major databases: PubMed, ScienceDirect, Google Scholar, and the Cochrane Library, using predefined search strings tailored to each platform. For ScienceDirect, the query was: ("OCT angiography" OR "OCTA" OR "optical coherence tomography angiography") AND ("glaucoma suspects" OR "preperimetric glaucoma" OR "glaucoma suspect"), with filters applied for research articles, open access content, and publication years between 2014 and 2025. Google Scholar was queried using a similar string but limited to English-language publications from 2014 to 2025, and screening was restricted to the first 100 results sorted by relevance. The Cochrane Library search employed the same keywords and was restricted to publications from 2015 to 2025 in English, excluding inaccessible full texts. Finally, the PubMed search used the terms "OCT angiography," "OCTA," or "optical coherence tomography angiography" in combination with "glaucoma suspects," "preperimetric glaucoma," or "glaucoma suspect" in the title or abstract fields. Filters included free full-text availability, study types limited to observational studies, randomized controlled trials, or meta-analyses, and results were sorted by best match, with screening limited to the first 100 records.

Study Selection Process

**Figure 1:**
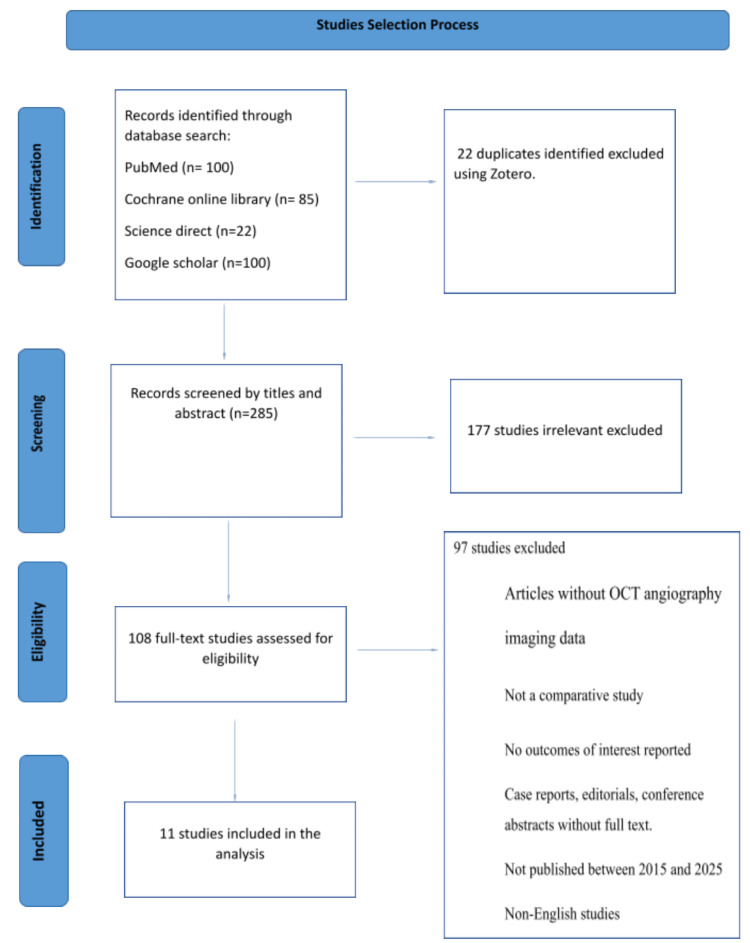
PRISMA flow diagram adapted to scoping reviews. OCT: optical coherence tomography

Two independent reviewers performed the study selection process in accordance with PRISMA-ScR (scoping reviews) reporting guidelines. Titles and abstracts of all studies identified through the initial search were screened, and potentially eligible articles were selected for full-text review. Full texts were then assessed for inclusion based on predefined eligibility criteria, and studies were included only when agreement was reached. Data extraction was conducted using a standardized form, and any discrepancies were resolved through discussion or consultation with a third reviewer when necessary. Quality assessment of the included studies was conducted by two independent reviewers. The study selection process is summarized in the PRISMA flow diagram (Figure 1).

Data Extraction

For this systematic review, data extraction was performed independently by two reviewers using a standardized form. Discrepancies were resolved through discussion or consultation with a third reviewer if needed. The process adhered to PRISMA-ScR guidelines, ensuring reproducibility and accuracy. The data extracted from the included studies are shown in Table 1.

**Table 1:** Data and key findings extracted from included studies OCT: optical coherence tomography; POAG: primary open-angle glaucoma; wiVD: whole-image vessel density; pfVD: perifoveal vessel density; mGCC: macular ganglion cell complex; cpVD: circumpapillary vessel density; RNFL: retinal nerve fiber layer; VF: visual field; SD-OCT: spectral-domain optical coherence tomography; GCC: ganglion cell complex; PPG: preperimetric glaucoma; AUROC: area under the receiver-operating characteristic curve; CCT: central corneal thickness; LC: lamina cribrosa; IOP: intraocular pressure; MRW: minimum rim width; GCIPL: ganglion cell-inner plexiform layer; ONH: optic nerve head; LC: lamina cribrosa; MS: mean sensitivity Key design elements, sample populations, imaging approaches, comparators, and major findings related to the diagnostic utility of OCTA in glaucoma suspects.

Study (Author, Year)	Design	Sample	Population	Intervention	Comparator	Outcomes	Key Findings
Akil et al., 2017 [3]	Prospective observational	56 eyes	Normal, preperimetric, mild POAG	OCTA vessel density (optic nerve head, peripapillary, papillary)	Healthy, preperimetric	Vessel density, RNFL, VF	Vessel density decreases stepwise from normal to mild POAG, strongly correlated with RNFL.
Hou et al., 2019 [8]	Cross-sectional	287 eyes	Healthy, preperimetric, early POAG	Macular vessel density & GCC thickness (OCTA + OCT)	Healthy	% loss in GCC & VD, AUROC	Vessel density loss occurs early; OCTA and OCT are equally effective for early detection.
Hou et al., 2018 [7]	Cross-sectional	153 subjects	Healthy, glaucoma suspect, glaucoma	Inter-eye VD & thickness asymmetry (OCTA, SD-OCT)	Healthy	VD & thickness asymmetry, AUROC	VD asymmetry is higher in suspects; useful for early screening.
Shoji et al., 2017 [2]	Longitudinal cohort	100 eyes	Healthy, suspect, POAG	Longitudinal VD loss vs. GCC change (OCTA, SD-OCT)	Healthy, suspect	Rate of VD & GCC change	Faster VD loss in POAG; OCTA detects changes before structural loss.
Suh et al., 2020 [9]	Cross-sectional	94 eyes	Preperimetric POAG	Deep-layer dropout in βPPA (OCTA)	With vs. without dropout	Dropout presence, RNFL, VF, LC defect	Dropout is linked to worse VF, RNFL thinning, and LC defects; it is an early marker of ONH damage.
Wang et al., 2020 [10]	Observational (case series)	79 eyes	Healthy, PPG, early PG	Macular VD & GCC thickness (OCTA)	Healthy	% reduction in VD & GCC, AUROC	Stepwise VD and GCC thinning; perifovea more affected in early glaucoma.
Yarmohammadi et al., 2016 [4]	Cross-sectional	153 eyes	Healthy, suspect, POAG	cpVD, wiVD, RNFL (OCTA & SD-OCT)	Healthy, suspect, POAG	VD, RNFL, VF MD	VD correlates more with VF damage than RNFL or rim area; independent predictor.
Yarmohammadi et al., 2017 [5]	Cross-sectional	86 eyes	OAG with hemifield VF defect, healthy	cpVD, pfVD, RNFL, mGCC (OCTA & SD-OCT)	Affected vs. intact hemiretina	VD, RNFL, mGCC, VF MS	VD reduced in both affected and intact regions; detects early glaucomatous damage.
Yarmohammadi et al., 2018 [6]	Cross-sectional	66 eyes	POAG with unilateral VF loss, healthy	wiVD, cpVD, pfVD, RNFL, mGCC, rim (OCTA & SD-OCT)	Unaffected POAG vs. healthy	Inter-eye VD & structure, AUROC	VD differences detected in unaffected eyes; higher AUROC than structural parameters.
Moghimi et al., 2018 [1]	Prospective longitudinal cohort	132 eyes (83 patients)	Mild to moderate POAG (mean follow-up ~27 months)	OCTA-derived macular (m-wiVD) and ONH vessel density (onh-wiVD); RNFL measured with SD-OCT	Eyes with higher vs. lower baseline m-wiVD and onh-wiVD	RNFL thinning rate; m-wiVD, onh-wiVD, CCT, MRW, GCIPL, IOP	Lower baseline m-wiVD/onh-wiVD significantly predicted faster RNFL loss. Each 1% drop in m-wiVD = 0.09 mm/year faster RNFL loss. CCT is also an independent predictor. Associations were statistically significant but weak (r² < 0.13). OCTA VD may help predict glaucoma progression.

Quality Assessment

Since the included studies were not randomized, their risk of bias was assessed using the ROBINS-I tool, as shown in Table 2. Although not mandatory in scoping reviews according to PRISMA-ScR guidelines, quality assessment was included to improve the interpretability of findings and to better contextualize the strength of the available evidence.

**Table 2:** Quality assessment of included studies using ROBINS-I tool Risk of bias evaluation across seven domains for each included study using the ROBINS-I tool. ROBINS-I: Risk Of Bias In Non-randomized Studies - of Interventions Note: Bias due to deviations from intended interventions was marked Not Applicable in all studies, as none involved an assigned intervention.

Study	Confounding	Selection of Participants	Classification of Interventions	Deviations from Intended Interventions	Missing Data	Measurement of Outcomes	Reported Results
Akil et al., 2017 [3]	Moderate	Low	Low	Not applicable	Low	Low	Low
Hou et al., 2019 [8]	Moderate	Low	Low	Not applicable	Low	Low	Low
Hou et al., 2018 [7]	Moderate	Low	Low	Not applicable	Low	Low	Low
Moghimi et al., 2018 [1]	Low	Low	Not applicable	Not applicable	Low	Low	Low
Shoji et al., 2017 [2]	Moderate	Low	Low	Not applicable	Low	Low	Low
Suh et al., 2020 [9]	Moderate	Low	Moderate	Not applicable	Low	Low	Low
Wang et al., 2020 [10]	Moderate	Low	Low	Not applicable	Low	Low	Low
Yarmohammadi et al., 2016 [4]	Moderate	Low	Low	Not applicable	Low	Low	Low
Yarmohammadi et al., 2017 [5]	Moderate	Low	Low	Not applicable	Low	Low	Low
Yarmohammadi et al., 2018 [6]	Low	Low	Low	Not applicable	Low	Low	Low

Data Synthesis

While there is a general consensus that OCTA can detect microvascular changes in glaucoma, the significant methodological heterogeneity, differing patient characteristics, and some conflicting findings across these studies suggest that a reliable and meaningful meta-analysis was not feasible. As an alternative, findings were synthesized narratively. Key outcomes (VD, diagnostic accuracy) were grouped thematically. Consistent patterns (e.g., reduced VD in suspects) were identified, while variations were explained by methodological differences. Higher-quality studies were prioritized to draw evidence-based conclusions about OCTA's role in detecting subclinical glaucoma changes.

Results

Study Selection

The study selection process began with 307 identified records. After removing 22 duplicates, 285 records were screened by title/abstract, excluding 177 irrelevant studies. Full-text review of 108 articles excluded 97 more (Not a comparative study, no outcomes of interest reported, Case reports, editorials, conference abstracts without full text, not published between 2015 and 2025, and non-English studies). Final inclusion comprised 11 studies meeting all eligibility criteria, following PRISMA-ScR guidelines with consensus resolution of disagreements.

Narrative Synthesis of Methodological Features

Nonetheless, the quality assessment is not mandatory for scoping reviews; it is approached to aid the interpretation of the findings. The overall quality assessment of the included studies reveals generally low risk of bias in most domains, with the primary concern being moderate risk of bias due to confounding. Most studies demonstrated low bias in participant selection, classification of interventions (where applicable), handling of missing data, outcome measurement, and reporting of results (see Table 2). However, confounding remains a recurring limitation, as many studies could not fully adjust for variables such as glaucoma medications, systemic conditions, or imaging quality. Notably, all studies were observational and thus had no deviations from intended interventions, rendering that domain not applicable. Despite these limitations, the methodological rigor in selection criteria, imaging protocols, and outcome reporting enhances confidence in the reported findings.

Synthesis of Findings

VD in glaucoma suspects and PPG: OCTA has proven to achieve substantial success in identifying microvascular changes that are subclinical among adults who present themselves as glaucoma suspects or PPG, and in most cases may even advance or complete the routine methods of diagnosis [3, 8, 10]. Glaucoma suspects are patients with risk factors or suspicious ocular manifestations without clearly established flaws in the VF, whereas PPG patients show an optic disc reduction or damage that has no typical VF loss [3]. The VD (whole image) in glaucoma suspects was 51.3% as compared to the 55.5% found among healthy eyes [4]. In eyes with a unilateral loss of the VF, the wiVD monitoring of the healthy eye (assigned as preperimetric) was on average 52.0% (significantly different than the value of 55.9 % in healthy eyes, P<0.001) [6]. This particular parameter on the wiVD was found to display the best diagnostic sensitivity (AUROC=0.84), compared to structural measures of macular GCC (0.78) and RNFL thickness (0.77) in differentiation between these unaffected eyes and healthy controls [6]. This indicates that OCTA detects early glaucomatous damage earlier than there are losses found in the standard visual fields [5, 6].

Diagnostic accuracy compared to structural OCT: When it comes to the question of diagnostic accuracy, ONH VD and RNFL thickness have been reported to present an analogous diagnostic precision when classifying healthy and glaucomatous eyes [1]. Moreover, it has also been demonstrated that the macular VD is lower in glaucomatous eyes than in healthy ones. The mean baseline macular whole-image VD was 50.0% +/- 3.7% and ONH whole-image VD was 52.4% +/- 3.7% in the glaucoma patients recruited in the study [1].

Measurements of VD on OCTA are also evidently different when compared to healthy controls in such early stages of glaucoma. As an example, the peripapillary VD, the optic nerve head VD, and the papillary VD are much higher in the control eyes compared to the preperimetric-glaucoma eyes, with the p-values of 0.001, 0.003, and 0.007, respectively [3]. It was identified that global and regional GCC VD was higher in healthy eyes compared to preperimetric eyes (all p-values 0.001) [8]. In particular, wiVD in PPG eyes was significantly decreased because it was 46.9% +/- 3.6% in PPG eyes and 50.9% +/- 2.8% in healthy eyes [10]. Moreover, deep-layer microvasculature dropout, that is, a disappearance of the microvasculature as a whole, was found in a significant proportion of preperimetric POAG eyes, that is, in 35.1% (33 of 94) of such cases [9]. This attrition was linked with a lesser mean deviation of VF and thinner RNFL [9].

Microvascular asymmetry as an early marker: When comparing OCTA to standard structural measurements like GCC thickness, studies indicate similar diagnostic accuracy for early glaucoma detection. For example, both GCC thickness and macula VD showed comparable diagnostic accuracy for discriminating preperimetric or early glaucoma eyes from healthy eyes (all P>0.05) [8]. In PPG eyes, the AUROC for wiVD was 0.824 (95% CI: 0.701-0.912), comparable to GCC thickness at 0.881 (95% CI: 0.768-0.952; P>0.05) [10]. Notably, inter-eye VD asymmetry parameters are significantly higher in glaucoma suspects (peripapillary 2.0% vs. 1.1%, P=0.014; macular 2.5% vs. 1.2%, P=0.027) compared to healthy individuals, whereas structural thickness asymmetries showed no significant differences [7]. This suggests that microvascular asymmetry may be an earlier indicator [7]. These findings are supported by Penteado et al., who found that OCTA measurements of macular vascular density were significantly associated with sensitivity losses in the central 10-2 VF, reinforcing the clinical utility of OCTA in central vision assessment [11].

Longitudinal changes in VD: Longitudinal studies further highlight OCTA’s utility, showing that the rate of macula VD loss is significantly faster in POAG eyes (−2.23%/yr) than in glaucoma suspects (0.87%/yr, P=0.001) or healthy eyes (0.29%/yr, P=0.004), even when GCC thickness changes are not yet significant [2]. This indicates that OCTA can detect progressive microvascular changes even in eyes without detectable structural thinning, suggesting its potential for earlier disease monitoring [2].

Discussion

OCTA effectively detects subclinical microvascular changes in glaucoma suspects and PPG. Key findings consistently show reduced VD; for example, wiVD is significantly lower in these groups compared to healthy eyes, with deep-layer microvasculature dropout also observed in preperimetric cases. Clinically, OCTA is crucial for early glaucoma detection, identifying these changes before standard VF defects manifest. Its ability to detect faster rates of VD loss prior to significant structural thinning suggests potential for earlier disease monitoring.

Regarding comparison to structural OCT, OCTA VD measurements often show diagnostic accuracy comparable to or even surpassing macular GCC and RNFL thickness for early detection. Furthermore, inter-eye VD asymmetry may be an earlier indicator than structural thickness asymmetries. Limitations include significant methodological heterogeneity across studies, differing patient characteristics, and confounding variables. Many of the included studies were observational in nature, and unmeasured systemic influences or imaging variability may have impacted VD measurements. Additionally, the use of different OCTA devices, segmentation protocols, and image processing parameters across studies may have contributed to variations in results and limited direct comparability. The sources do not identify a lack of longitudinal studies as a limitation, but highlight the utility of existing ones.

Future directions necessitate standardized protocols to address current heterogeneity and enable more robust comparative analyses. There is also a continuous need for long-term outcome studies to further establish OCTA’s predictive value in glaucoma progression. The main focus of this scoping review was to map the range of available evidence rather than perform a detailed risk of bias or quantitative synthesis.

## Conclusions

OCTA shows significant effectiveness in detecting subclinical microvascular changes in glaucoma suspects and PPG. After mapping the evidence, the key findings consistently indicate reduced VD, such as lower wiVD in these groups compared to healthy eyes, and even deep-layer microvasculature dropout in preperimetric cases. These changes can precede traditional visual field defects and even structural thinning detected by standard OCT. OCTA’s diagnostic accuracy often rivals or surpasses that of macular GCC and RNFL thickness for early detection, with inter-eye VD asymmetry potentially serving as an earlier indicator than structural asymmetries. Despite its promise, methodological heterogeneity across studies and differing patient characteristics highlight the need for further validation. Future efforts must focus on establishing standardized protocols and conducting comprehensive, long-term outcome studies to fully confirm OCTA's predictive value in glaucoma progression.

## References

[1] Moghimi S, Zangwill LM, Penteado RC (2018). Macular and optic nerve head vessel density and progressive retinal nerve fiber layer loss in glaucoma. Ophthalmology.

[2] Shoji T, Zangwill LM, Akagi T (2017). Progressive macula vessel density loss in primary open-angle glaucoma: A longitudinal study. Am J Ophthalmol.

[3] Akil H, Huang AS, Francis BA, Sadda SR, Chopra V (2017). Retinal vessel density from optical coherence tomography angiography to differentiate early glaucoma, pre-perimetric glaucoma and normal eyes. PLoS One.

[4] Yarmohammadi A, Zangwill LM, Diniz-Filho A (2016). Relationship between optical coherence tomography angiography vessel density and severity of visual field loss in glaucoma. Ophthalmology.

[5] Yarmohammadi A, Zangwill LM, Diniz-Filho A (2017). Peripapillary and macular vessel density in glaucoma patients with single-hemifield visual field defect. Ophthalmology.

[6] Yarmohammadi A, Zangwill LM, Manalastas PI (2018). Peripapillary and macular vessel density in patients with primary open-angle glaucoma and unilateral visual field loss. Ophthalmology.

[7] Hou H, Moghimi S, Zangwill LM (2018). Inter-eye asymmetry of optical coherence tomography angiography vessel density in bilateral glaucoma, glaucoma suspect, and healthy eyes. Am J Ophthalmol.

[8] Hou H, Moghimi S, Zangwill LM (2019). Macula vessel density and thickness in early primary open-angle glaucoma. Am J Ophthalmol.

[9] Suh MH, Na JH, Zangwill LM, Weinreb RN (2020). Deep-layer microvasculature dropout in pre-perimetric glaucoma patients. J Glaucoma.

[10] Wang Y, Xin C, Li M, Swain DL, Cao K, Wang H, Wang N (2020). Macular vessel density versus ganglion cell complex thickness for detection of early primary open-angle glaucoma. BMC Ophthalmol.

[11] Penteado RC, Zangwill LM, Daga FB (2018). Optical coherence tomography angiography macular vascular density measurements and the central 10-2 visual field in glaucoma. J Glaucoma.

